# Emerging and anticipated innovations in prostate cancer MRI and their impact on patient care

**DOI:** 10.1007/s00261-024-04423-4

**Published:** 2024-06-14

**Authors:** Eduardo Thadeu de Oliveira Correia, Atallah Baydoun, Qiubai Li, Daniel N. Costa, Leonardo Kayat Bittencourt

**Affiliations:** 1 Department of Radiology, University Hospitals Cleveland Medical Center, Cleveland, OH USA; 2 Department of Radiation Oncology, University Hospitals Cleveland Medical Center, Cleveland, OH USA; 3 Department of Radiology, University of Texas Southwestern Medical Center, Dallas, TX USA; 4 Department of Radiology, Case Western Reserve University, 11100 Euclid Ave, Cleveland, OH 44106 USA

**Keywords:** Artificial intelligence, Magnetic resonance imaging, Positron emission tomography, Prostatic neoplasms

## Abstract

Prostate cancer (PCa) remains the leading malignancy affecting men, with over 3 million men living with the disease in the US, and an estimated 288,000 new cases and almost 35,000 deaths in 2023 in the United States alone. Over the last few decades, imaging has been a cornerstone in PCa care, with a crucial role in the detection, staging, and assessment of PCa recurrence or by guiding diagnostic or therapeutic interventions. To improve diagnostic accuracy and outcomes in PCa care, remarkable advancements have been made to different imaging modalities in recent years. This paper focuses on reviewing the main innovations in the field of PCa magnetic resonance imaging, including MRI protocols, MRI-guided procedural interventions, artificial intelligence algorithms and positron emission tomography, which may impact PCa care in the future.

**Supplementary Information:**

The online version contains supplementary material available at 10.1007/s00261-024-04423-4.

## Introduction

Prostate cancer (PCa) remains the leading malignancy affecting men, with a population of over 3 million men living with the disease, and an estimated 288,000 new cases and almost 35,000 deaths in 2023 in the United States alone [1]. The high prevalence of PCa has led to the establishment of comprehensive diagnostic and treatment strategies, which, while essential, also pose a significant financial burden on healthcare systems, with an estimate of over 22 billion dollars being spent on PCa care in the United States, in 2022 [2]. Over the last few decades, imaging has been a cornerstone in PCa care, playing a crucial role in the detection, staging of PCa, assessment of PCa recurrence, or even guiding diagnostic or therapeutic interventions.

**Fig. 1:**
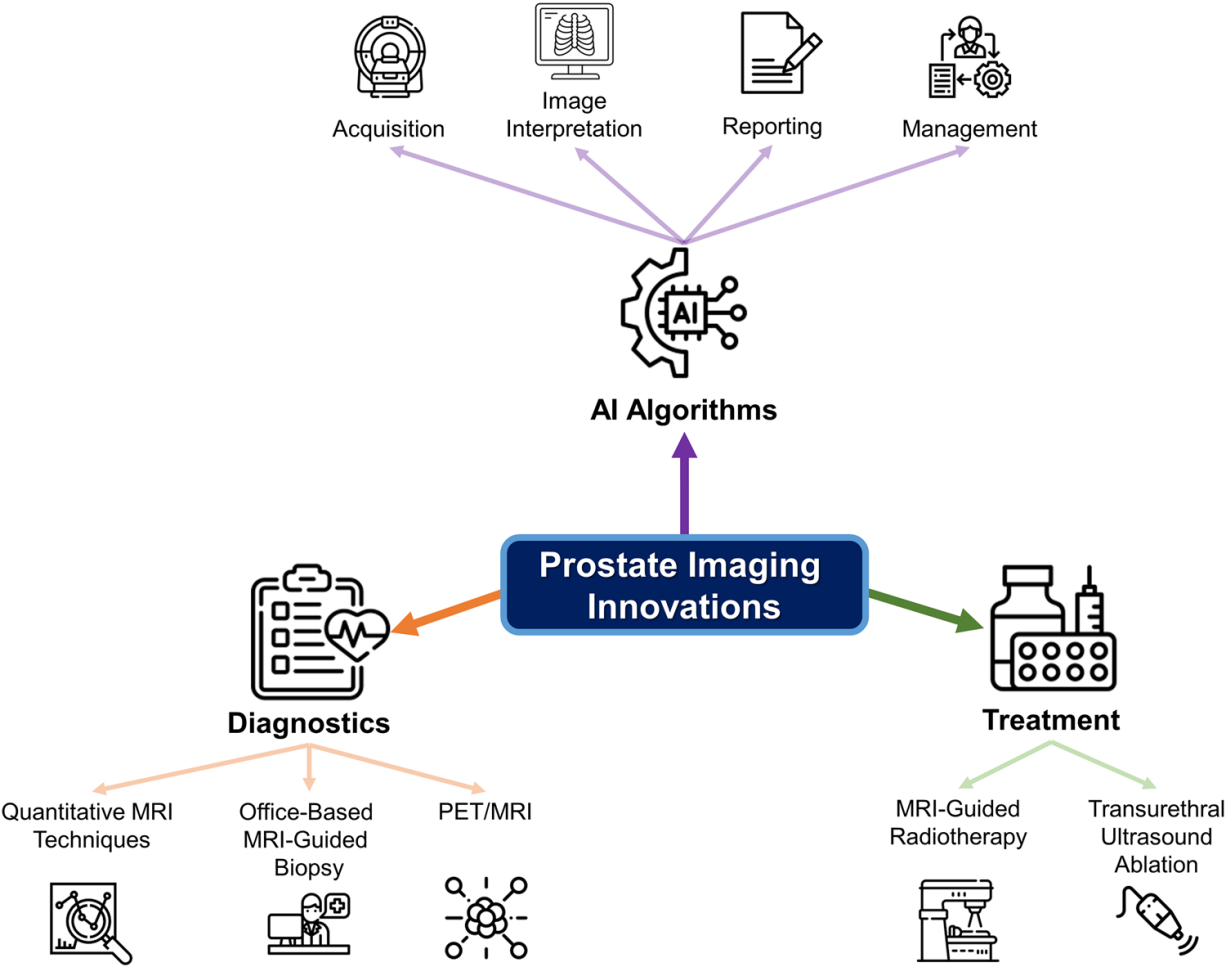
Innovations in the field of prostate imaging. Office-based MRI-guided biopsy refers to a low-field MRI equipment that was designed for urologists and radiologists to perform MRI-guided biopsies within the office. MRI – magnetic resonance imaging; PET – positron emission tomography

Remarkable advancements have been made in the field of imaging modalities in recent years, in order to improve diagnostic accuracy and overall outcomes of PCa. This is exemplified by the significant development of magnetic resonance imaging (MRI), positron emission tomography (PET), and artificial intelligence (AI) algorithms, as detailed in Fig. 1. Since it is not possible to cover every novelty in the broad field of PCa imaging, this paper focuses on reviewing the main innovations in PCa MRI, including MRI protocols, MRI-guided procedural interventions, artificial intelligence algorithms, and PET, which may impact PCa care in the future.

## Diagnostic magnetic resonance imaging

### Quantitative magnetic resonance imaging protocols

Over the past decade, a myriad of quantitative prostate MRI techniques have emerged, particularly to provide radiologists objective and reproducible MRI-derived tissue parameters to base diagnostic decisions. For instance, quantitative techniques have shown interesting results in enhancing the diagnostic capabilities of prostate MRI. This is the case with imaging methods such as Restriction Spectrum Imaging (RSI), Hybrid Multidimensional MRI (HM-MRI), Luminal Water Imaging (LWI), Vascular, Extracellular, and Restricted Diffusion for Cytometry in Tumors (VERDICT), and Magnetic Resonance Fingerprinting (MRF). This section as well as Table 1 detail the main features of quantitative prostate MRI techniques, exploring how they may impact clinical care. Hereby we also review the challenges currently limiting the implementation of quantitative MRI techniques into PCa diagnostic care.

**Table 1:** Characteristics, current applications and limitations of quantitative prostate magnetic resonance imaging techniques. ADC – apparent diffusion coefficient; AUC – area under the curve; csPCa – clinically significant prostate cancer; iPCa – insignificant prostate cancer; min – minutes; mpMRI – multiparametric magnetic resonance imaging; MRI – magnetic resonance imaging; PCa – prostate cancer; TE – echo time

Technique	Purpose	Current Evidence	Acquisition time	Limitations
Hybrid-Multidimensional MRI	Measures the change in T2 and ADC in response to changes to TE and b-value	Excellent agreement with histopathology in quantifying stroma, epithelium, and lumen [4] Can improve the specificity and accuracy of less-experienced readers to detect csPCa, increasing interreader agreement when combined with mpMRI [5] When used alone has a similar AUC to detect csPCa to mpMRI and a mean interpretation time 71% lower than of conventional mpMRI [6]	8–12 min [4, 6]	Lack of multicenter, prospective studies
Luminal Water Imaging	Measure the fraction of the longer T2 curve, which was demonstrated to strongly correlate with the amount of luminal space in the prostatic tissue	Luminal water fraction was associated with Gleason scores and had an excellent AUC for PCa detection [11] The combination of multiple luminal water index-derived parameters were also able to differentiate GG ≤ 2 and GG ≥ 3 PCa, with a higher AUC than ADC and PI-RADS scoring criteria alone [12]	~ 10 min [13]	Lack of larger, multicenter prospective studies
MR Fingerprinting	Simultaneous acquisition of multiple tissue properties by varying acquisition parameters in a pseudorandom manner	Was able to differentiate normal prostatic tissue from csPCa in both peripheral and transition zones, with exceptional reproducibility [17–19] Reference values for the normal peripheral zone available in the literature [20]	~ 10 min [20]	Lack of multicenter, prospective studies validating its real-world clinical application
Restriction Spectrum Imaging	Estimates the individual contributions of distinct tissue compartments to diffusion signal	Significantly improved the AUC for csPCa detection compared with PI-RADS scores alone [25]	~ 5 min [22, 23]	Lack of multicenter, prospective studies Relies solely on diffusion signal
VERDICT	Characterize tissue properties by acquiring diffusion-based signals from water in cells, in the vascular network, and in the interstitium	Was shown to better differentiate csPCa from iPCa or benign prostatic tissue compared with conventional ADC maps [28, 29]	~ 12 min [28]	Lack of multicenter, prospective studies using different scanners

### Hybrid multidimensional magnetic resonance imaging

**Fig. 2:**
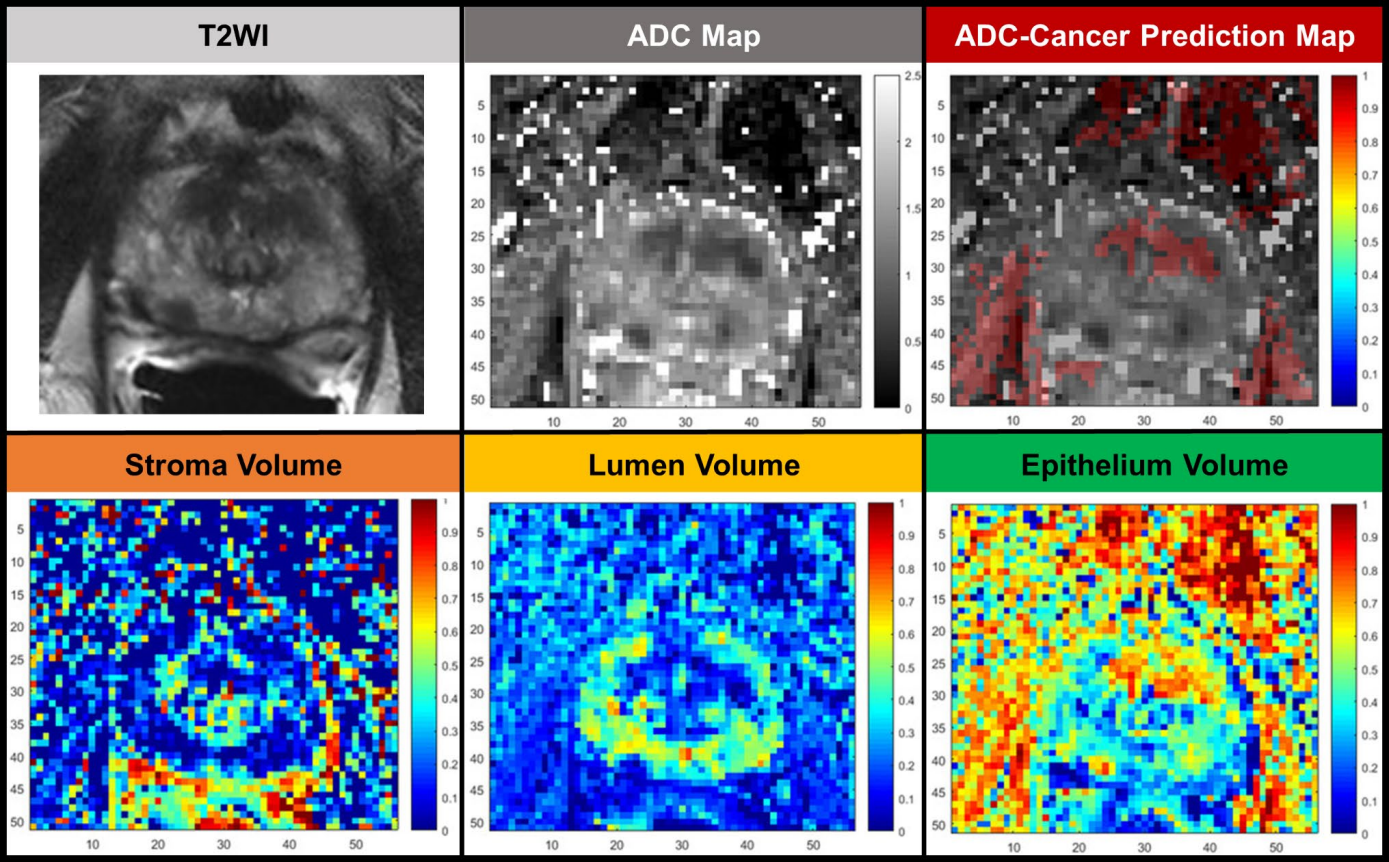
A 75-year-old man which underwent a standard MRI examination with the addition of hybrid multidimensional MRI for a prospective clinical trial. The bottom row depicts maps of fraction of stroma, lumen and epithelium volume, which were shown to strongly correlate with their fractions in whole mount prostate pathology [4]. Through a predicted cancer map generated by hybrid multidimensional MRI overlaid over an ADC map (third column, first row), it was possible to detect a lesion in the anterior apex of the prostate that was initially missed by a radiologist who read the standard MRI examination. This lesion was biopsied and results revealed a Grade Group 2 cancer. Image courtesy of Dr. Aritrick Chatterjee and Dr. Aytekin Oto from the University of Chicago, Illinois

Hybrid Multidimensional MRI (HM-MRI) is a novel MRI technique that can characterize tissue microstructure, by measuring the change in T2 and ADC in response to changes to echo time and b-value [3]. In the field of prostate imaging, HM-MRI was capable of quantifying the amount of lumen, stroma, and epithelium of prostate glands in-vivo, with excellent agreement with quantitative ex-vivo histologic evaluation (Fig. 2) [4]. Moreover, a previous retrospective study demonstrated that HM-MRI can improve the specificity and accuracy of less-experienced readers to detect csPCa, increasing interreader agreement when combined with multiparametric MRI (mpMRI) [5]. Another study on the same patient population showed that HM-MRI alone had a similar area under the curve to detect csPCa to mpMRI. Beyond that, the mean interpretation time of HM-MRI was 71% lower than that of conventional mpMRI [6]. Additionally, measurements of prostate tissue composition and the area under the curve to differentiate PCa from benign prostate tissue were similar between scanners of two vendors [7]. In a preliminary analysis of 92 patients of an ongoing clinical trial, HM-MRI also appeared to have higher sensitivity and accuracy than mpMRI on a per-sextant basis [8]. Although this is an important step toward the future of the virtual assessment of prostate histology, acquisition times for HM-MRI are still long (8–12 min on average) [4, 6]. Also, larger studies performed across scanners from different vendors are still needed to validate the application of HM-MRI in clinical practice.

### Luminal water imaging

Luminal water imaging (LWI) is an application derived from multi-exponential T2 mapping [9]. This is based on the principle that T2 decay curves acquired from prostatic tissue are predominantly bi-exponential [10] and that a longer T2 could be due to water protons residing inside the lumen, whereas a shorter T2 could be attributed to water protons located inside stromal or epithelial components of prostatic tissue [9]. Therefore, LWI can measure the fraction of the longer T2, which was demonstrated to strongly correlate with the luminal space in prostatic tissue measured in whole-mount histology sections, named luminal water fraction [9]. The clinical application of LWI was demonstrated in a previous small prospective study that found that luminal water fraction had a significant correlation with Gleason scores and had an excellent AUC for PCa detection [11]. In another study, the combination of multiple LWI-derived parameters was shown to be able to differentiate grade group ≤ 2 and grade group ≥ 3 PCa, with a higher AUC than ADC and PI-RADS criteria [12]. Acquisition times for LWI are around 10 min [13] but studies have proposed solutions to shorten acquisition times [14, 15]. Still, although LWI has been tested in prospective investigations, these studies had a small sample size and their results need to be confirmed by larger cohorts.

### Magnetic resonance fingerprinting

**Fig. 3:**
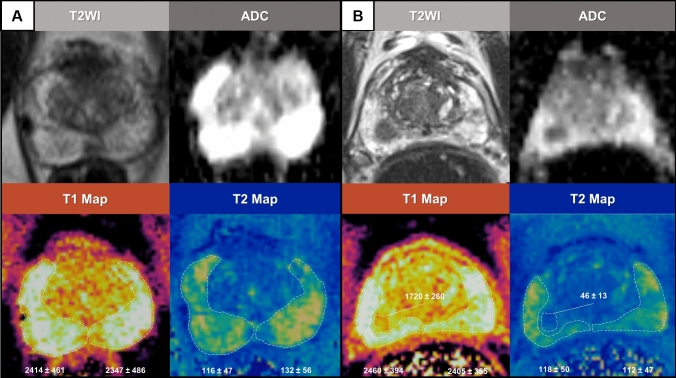
Magnetic resonance fingerprinting of the prostate. The case on the left **A** illustrates a PI-RADS 2 examination with a normal-appearing peripheral zone, with mean T1 of 2414 and 2347 ms for the right and left lobes of the peripheral zone and T2 relaxation times of 116 and 132 ms for the right and left lobes of the peripheral zone, respectively. The case on the right **B** illustrates a PI-RADS 4 examination. Regions of interest drawn on the peripheral zone demonstrate significantly higher T1 and T2 relaxation times of the normal-appearing peripheral zone compared with an annotated lesion in the right lobe of the peripheral zone (T1 and T2 values, respectively: lesion—1720 and 46 ms, right lobe of the peripheral zone—2460 and 118 ms, left lobe of the peripheral zone—2405 and 112 ms). This lesion was biopsied and results revealed a Grade Group 2 prostate cancer

Magnetic Resonance Fingerprinting (MRF) is an FDA-approved quantitative MRI technique capable of simultaneously quantifying multiple tissue parameters [16]. In the field of prostate imaging, MRF-derived T1 and T2 relaxation times combined with standard apparent diffusion coefficient (ADC) mapping were able to differentiate normal prostatic tissue from clinically significant prostate cancer (csPCa) in both peripheral and transition zones (Fig. 3) [17, 18]. Importantly, a multicenter study that investigated the repeatability and reproducibility of prostate MRF across several scanners in different continents demonstrated a low intra- and interscanner variation of T1 and T2 relaxation times [19]. MRF-derived T1 and T2 relaxation times of the normal peripheral zone of the prostate have also been comprehensively studied and could potentially be used as reference values for clinical studies [20]. Although ongoing studies are trying to accelerate acquisition times of prostate MRF, it currently takes around 40 s per slice to be acquired. This totalizes approximately 10 min to cover the whole prostate [20]. Nonetheless, further prospective studies incorporating MRF maps on clinical workflows of biopsy selection or PCa decision-making are still needed.

### Restriction spectrum imaging

Diffusion-weighted imaging (DWI) techniques are a crucial part of a PI-RADS compliant multiparametric prostate MRI protocol and serve as the dominant sequence for characterizing suspected peripheral zone (PZ) lesions as well as to upgrade transition zone (TZ) lesions to a higher PI-RADS category [21]. Restriction spectrum imaging (RSI) was developed as an advanced DWI technique that can be acquired in less than 5 min and estimates the individual contributions of different tissue compartments to the diffusion-weighted signal [22, 23]. Previous retrospective studies have investigated the role of an RSI-derived coefficient that is based on restricted intracellular water signal, named the RSI restriction score (RSI-RS), and showed that this score can improve the detection and characterization of PCa [23, 24]. Beyond that, a previous retrospective investigation has found that RSI-RS had a similar area under the curve (AUC) for detecting csPCa compared with PI-RADS scores, while it significantly improved the AUC for csPCa detection compared with PI-RADS scores alone [25].

### VERDICT

The VERDICT technique was developed as a method to characterize tissue properties by acquiring diffusion-based signals from water in cells, in the vascular network, and in the interstitium [26]. In a small study, VERDICT was shown to be able to discriminate benign regions in the prostate from PCa by pooling differences in cellular, vascular, and extracellular-extravascular space fractions [27]. In another study, intracellular volume fraction maps derived from the VERDICT framework were shown to better differentiate csPCa from iPCa or benign prostatic tissue compared with conventional ADC [28]. More recently, it was demonstrated that intracellular volume fraction maps had a better diagnostic performance to detect csPCa compared with both ADC and PSA-density [29]. Acquisitions times for VERDICT-MRI of the prostate are around 12 min [28]. Nevertheless, future multicenter studies using different scanners are still needed to confirm the reproducibility of these findings.

## MR-guided procedures

MR-guided biopsy and MR-transrectal ultrasound fusion biopsy are already established procedures, largely utilized to aid PCa clinical decision-making. Therefore, they are not covered in this section. In the past decades, several focal therapy strategies have been proposed as alternatives to radical prostatectomy and radiation therapy to treat PCa. This is the case of high-intensity focused ultrasound, cryo- and radiofrequency ablation, focal laser ablation, irreversible electroporation, focal brachytherapy, photodynamic therapy, and prostatic artery embolization [30]. Hereby we will discuss MR-guided or MR-based procedures that have recently started making an impact or hold promise in impacting PCa care in the future.

### Focal laser ablation

MRI-guided focal laser ablation (MRI-FLA) of the prostate is a minimally invasive technique that uses a laser to ablate the target prostatic tissue under MRI guidance. Real-time MRI thermometry is used to ensure accurate targeting and monitoring during the procedure. Several applicator positions are utilized to ensure adequate tumor ablation with margins. A bladder catheter is also employed for continuous drainage [31, 32]. Prior to the procedure, the prostate gland, targeted tumor, rectal wall, and urethra are contoured on T2WI manually. After that, a coaxial catheter is transperineally or transrectally inserted under MRI guidance together with a titanium obturator to the target region [31, 33]. Following that, the obturator is replaced with an optical fiber featuring a cylindrical diffusing tip [31, 33]. To confirm the correct position of the applicator, a sub-therapeutic power is applied, subsequently followed by the application of tissue-ablating energies, which are verified using real-time MRI thermometry [31, 33]**.** Following successful tissue ablation, a post-ablation MRI is performed [34]. Different studies have investigated the effectiveness of MRI-FLA for the treatment of localized PCa. An initial phase I trial showed that MRI-FLA was feasible, without notable changes in urinary or sexual function after 6 months of the procedure. However, among nine patients included in this first trial, two had Gleason grade 6 cancer after 6 months of the procedure, with both patients having partial ablation of the target zone when MRI images were retrospectively reviewed [35]. In another phase I trial including 15 patients, after 3 years of follow-up, seven patients had residual cancer proximal or adjacent to the target ablation zone and four had to undergo salvage treatment. Additionally, while urinary function and quality of life scores were similar before treatment and after 3 years of follow-up, the authors observed a significant decrease in sexual function scores [33]. In a Phase II trial including 27 patients, 26 had no PCa on MRI-guided biopsy of the ablation zone 3 months after MRI-FLA. However, at 12 months, ten patients had cancer identified on systematic biopsy, three of which were in the ablation zone. Regarding adverse events in this trial, hematuria was reported in four patients, perineal ecchymosis in three, and urinary retention in two. Urinary and sexual function scores were similar before and 12 months after the procedure, although the sexual function score was considerably lower than before the procedure at 1 and 3 months after MRI-FLA [36]. Another study comprised of 49 subjects who underwent MRI-FLA demonstrated that treatment was successful in 39 patients, whereas persistent cancer was found in ablated areas in ten patients, with all ten patients exhibiting incomplete ablation of the target zone. In this same study, no significant sexual, urinary, or bowel side effects were observed in the included patients 18 months after treatment [37]. Therefore, although MRI-FLA appears to be safe for treating localized PCa, effective ablation of target zones still needs to be further refined and long-term outcomes require further investigation.

### Transurethral ultrasound MR-guided ablation

**Fig. 4:**
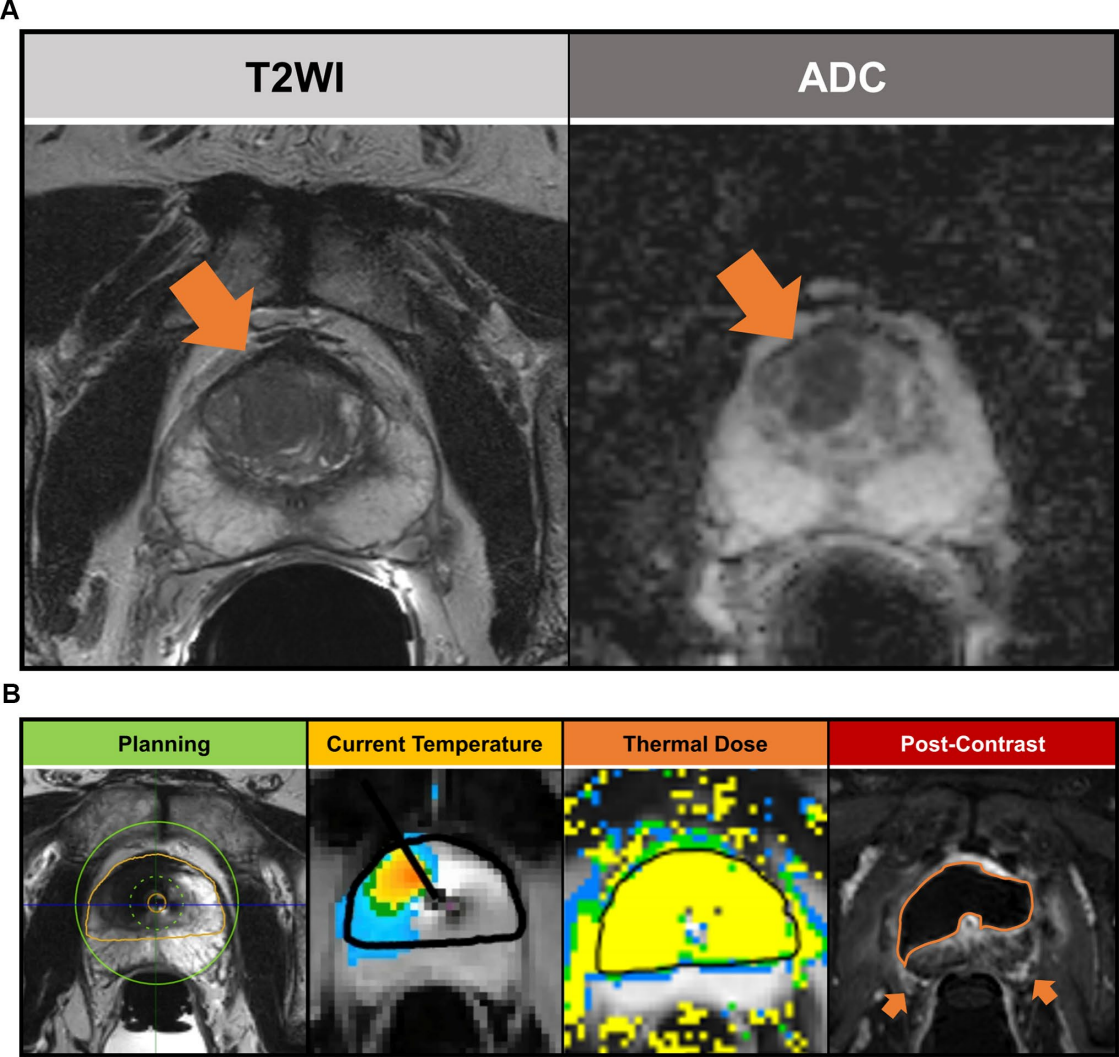
A 59-year-old man with elevated PSA (10.6 ng/mL) and fusion biopsy revealing grade group 2 cancer from a right mid-anterior transition zone PI-RADS 5 lesion (**A**), as shown by the axial T2-weighted (left) and the corresponding apparent diffusion coefficient map (right) images. The patient underwent MRI-guided transurethral ablation consisting of an anterior hemiablation (**B**). In the treatment planning phase (first image from the left), high-resolution axial T2-weighted images are used to draw the ablation zone (orange line). The image also demonstrates the maximum treatable volume (green circle) located 3 cm from the urethral applicator (small orange circle). Real-time MR thermometry images are obtained during the treatment (selected timepoint shown in the second image from the left, video available as supplemental material [S1]) ensuring adequate heating (yellow pixels) up to the periphery of the treatment zone. In addition to current temperature maps, the treatment console can generate thermal dose maps (third image from the left) providing a better delineation of the ablated areas. After ablation is complete, pre- and post-contrast T1-weighted images are obtained to determine the non-perfused volume and serve as a marker of the treatment quality. In this case, no enhancing tissue is noted in the area planned to be ablated (fourth image from the left). Note the safe distance between the ablation area and the neurovascular bundles (orange arrows). A follow-up biopsy performed 12 months after the ablation was negative for cancer

MRI-guided transurethral ultrasound ablation (TULSA) was introduced as a real-time MRI-guided procedure that ablates lesions through thermal ultrasound waves, while it cools the rectal and urethral wall to avoid tissue damage [38]. In a stepwise manner, the TULSA procedure begins with the placement of a urethral applicator that contains ultrasound elements and an endorectal cooling device to protect the anterior rectal wall [38]. The ultrasound applicator is then positioned to the desired location and high-resolution T2-weighted images are used to contour the ablation zone. Depending on the number and location of cancers, presence of lower urinary tract obstructive symptoms, and distance to the external urethral sphincter, bladder neck, and neurovascular bundles, the treatment plan can be customized from a truly focal to a whole gland treatment [38]. During treatment, real-time MR thermometry images from the entire gland are available to monitor the degree and extent of tissue heating (Fig. 4a, b). Objectively, a multicenter trial demonstrated that TULSA was effective in reducing PSA levels by ≥ 75% in 96% of included patients, with a rate of severe adverse events of 8%, including urethral stricture, genitourinary infection, urinary retention, urinary calculus, pain and urinoma, which resolved by the end of one year of follow-up [39]. Specifically, among men with grade group 2 PCa before intervention who underwent biopsy 12 months after the procedure, 79% were free of grade group 2 PCa [39]. At 5 years of follow-up, the median PSA was 0.6 ng/mL, compared with the baseline PSA of 6.3 ng/ml. Still, 22% of patients who underwent TULSA needed salvage treatment. Safety-wise, at 5 years, 92% of patients had pad-free continence, and 87% preserved erectile function [40]. Nonetheless, adequate patient selection for TULSA and the long-term effectiveness of focal therapy delivered by TULSA on hard outcomes remain to be established. The multi-center phase III CAPTAIN study is ongoing and will investigate if TULSA is non-inferior to radical prostatectomy regarding 3-year rates of freedom from treatment. This study will also investigate the safety of TULSA and secondary endpoints such as survival, complications, quality of life, biochemical failure, and post-operative recovery [41].

### Low-field office-based MRI-guided biopsy

The PROMAXO MRI system is an FDA-approved office-based low-field MRI equipment designed for urologists and radiologists to perform MRI-guided biopsies [42]. A prostate biopsy workflow using PROMAXO begins with the annotation of potential biopsy targets on T2WI obtained from a 3 T MRI scan, that is uploaded to the PROMAXO system [43]. For the biopsy procedure, the patient is positioned in a high-lithotomy position, their pelvic region is covered with surface coils and is positioned close to the center of the field of view of the PROMAXO MRI scan [43]. Following this, the imported T2WI images from the 3 T scan are registered in the PROMAXO T2WI scan by the physician performing the biopsy [43]. Finally, the physician selects the target location and obtains tissue samples through a transperineal biopsy [43]. Nonetheless, there is still a lack of studies comparing PROMAXO with standard MR-transrectal ultrasound fusion biopsy.

### Magnetic resonance imaging-based radiotherapy

Radiotherapy is one of the established cornerstone therapies for the management of localized PCa [44]. Still, the challenge of optimizing the delivery of radiation therapy to ensure PCa control, while avoiding damage to benign tissues remains [44]. In fact, MRI has transfigured the process of PCa external beam radiotherapy delivery through two perspectives: (1) MRI-based radiation therapy planning and (2) On-board MRI-guided radiation therapy.

Since the advent of three-dimensional conformal radiotherapy and later intensity-modulated radiation therapy, computed tomography (CT) has been traditionally used for planning target volumes (PTVs) and normal organs at risk delineation. While CT enables an exquisite discrimination capacity in many anatomical sites such as the thorax, it has a limited role in depicting prostate anatomy or glandular changes. Comparative contouring studies have shown that, compared to CT-based contouring, MRI allowed a reduction in tumor volumes and inter-observer variability [45]. More importantly, MRI offers the particular advantage of vessel-sparing PCa radiotherapy wherein MRI images are used to minimize dose delivery to the pudendal artery, potentially minimizing post-radiation therapy sexual dysfunction [46]. Finally, the use of MRI for radiation therapy planning has enabled the delivery of a focal boost to the prostate index lesion, which improved the biochemical disease-free survival in a phase III randomized trial [47].

**Fig. 5:**
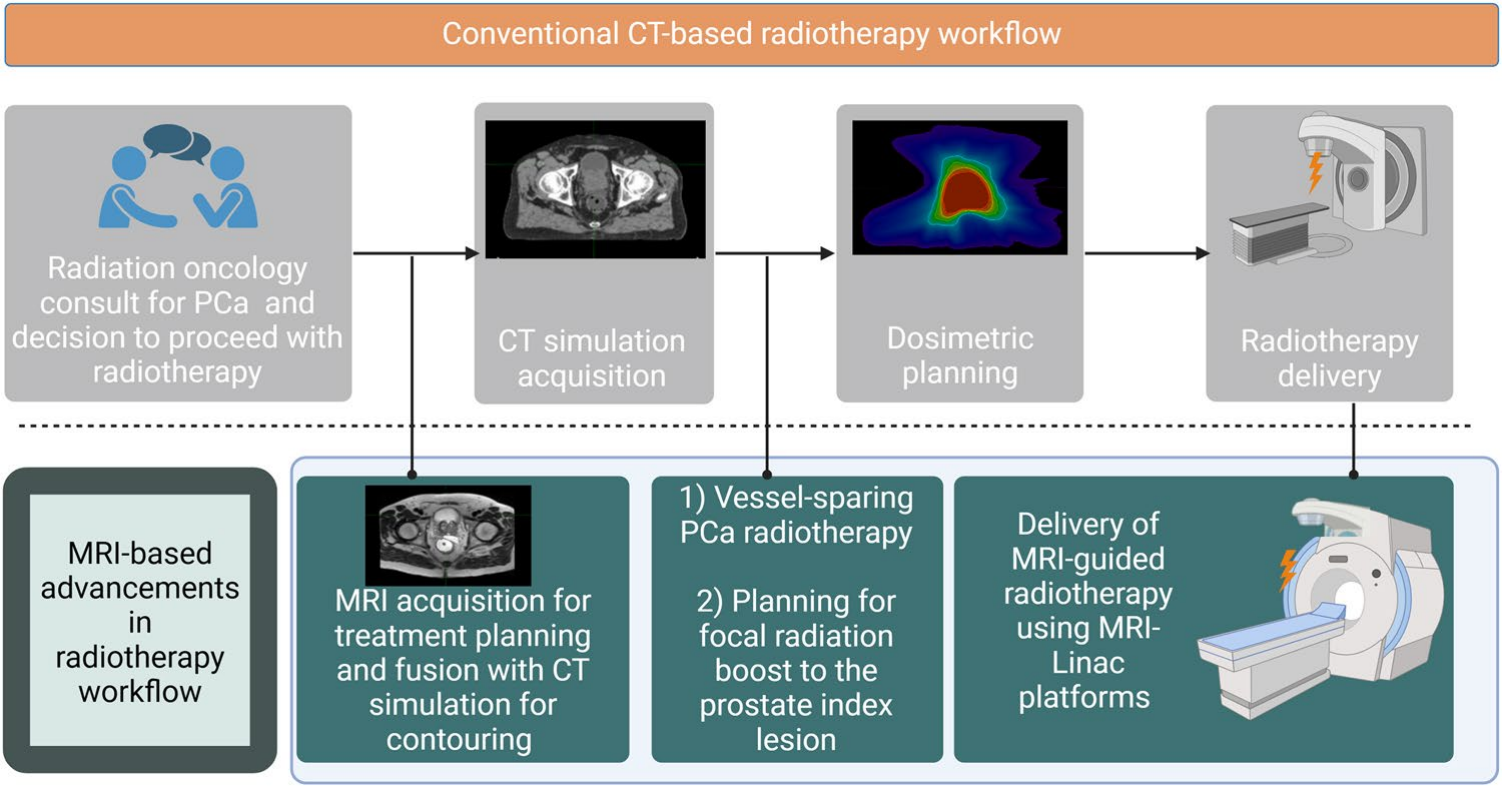
Conventional CT-based radiotherapy vs Advancements made by the addition of MRI to the radiotherapy workflow. PCa- prostate cancer

As for image-guided radiotherapy, it has been commonly performed through the acquisition of onboard cone beam computed tomography (CBCT) [48]. Nonetheless, CBCT has poor soft tissue discrimination capacity, and therefore invasive placement of radiopaque fiducials to serve as markers of prostate positioning is usually required [48]. Magnetic-resonance imaging-guided radiotherapy techniques using MRI-linac, have emerged to provide better malignant-benign tissue differentiation, account for organ deformation by the use of daily imaging, and reduce toxicity to normal tissues [48, 49]. Recent data from the phase 3 MRI-Guided Stereotactic Body Radiotherapy for Prostate Cancer (MIRAGE) randomized trial showed that, compared with CT-guided radiotherapy, MRI-guided radiotherapy reduced the decrease in quality of life and the incidence of both genitourinary and gastrointestinal toxicity at one-month post-procedure [50]. Nevertheless, studies with a longer follow-up period are still needed to evaluate the difference in the occurrence of late bowel or urinary complications between MRI- and CT-guided radiotherapy. Advancements made by MRI in the radiotherapy workflow are detailed in Fig. 5.

## Artificial intelligence in MRI

AI applications may facilitate or even improve prostate MRI acquisition, interpretation, and reporting, as well as guide the MRI-directed management of PCa.

For image acquisition, multiple AI algorithms were investigated both to improve acquisition times and to improve image quality. Gassenmaier et al. demonstrated, in a retrospective study of 30 patients, that a deep-learning T2WI TSE imaging protocol was 65% faster compared to conventional T2WI TSE imaging. At the same time, the authors showed that the deep-learning T2WI protocol generated images with superior image quality when assessed by two different radiologists using a Likert score [51]. Ueda et al. also demonstrated that a deep learning algorithm for the reconstruction of DWI images increased the signal-to-noise ratio, the contrast-to-noise ratio and had superior qualitative image quality compared with standard reconstructions, while they did not affect the quantitation of ADC values [52]. However, deep-learning acquisitions and reconstruction techniques still need to be further validated to be adopted in radiology practice. This was exemplified by van Lohuizen et al., which showed that although deep-learning accelerated T2WI reconstructions produced images with appropriate visual quality, the diagnostic performance of deep-learning reconstructions was lower than original T2WI images [53]. There has also been a growth in the interest of assessing prostate MRI quality in the last few years, particularly after the publication of the PI-QUAL score [54]. Lin et al. and Belue et al. developed and investigated the performance of a deep-learning tool to assess prostate MRI T2WI quality [55, 56]. This tool was shown to have an accuracy of 85% for classifying prostate T2WI images binary as of non-diagnostic quality or acceptable/optimal diagnostic quality when compared to an experienced abdominal radiologist [56]. Additionally, in another study, the scans scored as high-quality had significantly higher targeted-biopsy cancer detection rates for PI-RADS 4 lesions, when compared to low-quality T2WI [55]. Other groups have also demonstrated that deep-learning algorithms to assess the image quality of bi-parametric prostate MRI are feasible. Still, the agreement between this deep-learning algorithm and expert assessment was fair to good [57]. Future studies should focus on improving the performance of AI models that can be easily integrated into the PACS system, to evaluate the quality of bi-parametric and multiparametric prostate MRIs.

Within image interpretation, several studies have focused on developing different deep-learning algorithms for prostate segmentation. In these studies, deep-learning algorithms had a high mean dice similarity coefficient, ranging from 0.88 to 0.93, compared with manual segmentation of the whole prostate performed by trained radiologists [58–60]. Several studies have also investigated the accuracy of deep learning and radiomics techniques to detect and characterize suspicious lesions. For instance, a previous meta-analysis that combined data from 12 studies showed that machine learning algorithms had an overall AUC of 0.86 to detect csPCa, compared with biopsy results (n = 9) or histopathological assessment of prostatectomy specimens (n = 3) [61]. More recently, Hamm et al. demonstrated an interactive deep learning algorithm that not only detects csPCa lesions at bi-parametric MRI but also explains the imaging features on which lesion detection was based [62]. The authors showed that this algorithm had an AUC to detect csPCa of 0.87 and had an 80% accuracy in displaying visual and textual explanations of the findings compared to experts [62]. Additionally, readers who were assisted by the algorithm had an improvement in confidence in assessing PI-RADS 3 lesions and also had almost a one-minute reduction in MRI reading time compared with those who did not use the algorithm [62]. For lesion classification, a deep-learning algorithm was shown to have similar performance to residents and less-experienced radiologists for classifying lesions into PI-RADS categories, albeit it had a significantly worse performance compared with experienced readers [63].

Beyond image acquisition and interpretation, large language models have been increasingly popular in recent years and could potentially aid in facilitating the interpretation of radiology reports by the general population. In fact, Li et al. demonstrated that ChatGPT was able to simplify the language of radiology reports to 8th grade reading level, while also reducing their word count [64], which could facilitate the understanding of reports by the general population, particularly those with lower health literacy levels. Nonetheless, large language AI models still need to be further validated for their safe implementation in practice.

Regarding MRI-directed management of PCa, AI applications can also facilitate biopsy guidance and decision-making after prostate MRI. Although still in the initial stages, previous studies have shown AI tools that can perform real-time segmentation of the prostate during MR-transrectal ultrasound fusion biopsy, which could improve the selection of biopsy targets [65, 66]. AI-powered decision-making models incorporating data from conventional MRI scans, quantitative MRI sequences, and serum markers may also improve patient selection for prostate biopsy in the future.

Still, evidence on the performance of machine learning algorithms for the detection and classification of prostatic lesions is based on retrospective studies. Additional concerns limiting the broader implementation of AI models for prostate MRI encompass issues such as cost and a lack of transparency in both the AI algorithms and the underlying reasoning that informs the output of these models. Therefore, the successful integration of AI algorithms into clinical practice relies not only on their performance in larger, multi-center prospective studies but also on their cost-effectiveness and the level of trust they instill among radiologists. Explainable AI models are also expected to play a pivotal role in fostering trust in algorithms and placing radiologists at the forefront of the future of radiology practice, steering away from ineffective attempts to position AI algorithms as potential replacements for radiologists. These models will also shed light on the conventional perception of AI algorithms as opaque "black boxes" and help mitigate the occurrence of AI hallucinations. Position statements about the use of AI algorithms for the detection of clinically significant PCa from the PI-RADS committee are currently under development and may guide the real-world application of AI algorithms as well as identifying key limitations of current AI algorithms that warrant future investigations. Potential applications for artificial intelligence algorithms in the field of prostate imaging are described in Table 2.

**Table 2:** Potential applications for artificial intelligence algorithms in prostate imaging

Steps	Tasks
**Image Acquisition**	**Automated sequence acquisition and quality assessment**
	• Automatically identifying key anatomical landmarks to guide and refine the selection of imaging planes
	• Automated and reproducible assessment of image quality
**Image Interpretation**	**Organ Segmentation**
	• Precise delineation of prostate boundaries and compartments
	**Lesion Segmentation**
	• Automated identification and delineation of suspicious lesions
	**Lesion Detection**
	• Detection of subtle lesions, reducing false negative MRIs
	**PI-RADS Scoring**
	• Automated assignment of Prostate Imaging Reporting and Data System (PI-RADS) scores
	**Staging**
	• AI-driven assessment of tumor stage based on imaging features
**Reporting**	**Automated Reporting**
	• Generation of structured reports based on AI-driven analysis
	**Translation to Lay Language**
	• Summarization of complex findings in understandable language
**Management**	**Decision-Making Models**
	• Integration of radiomics, demographic data, and serum markers
	• Personalized risk assessment for informed clinical decision-making
	**Biopsy and Treatment Planning**
	• AI-guided recommendations for biopsy site selection
	• Treatment planning assistance for optimal therapeutic strategies

*AI* artificial intelligence, *MRI* magnetic resonance imaging

## Positron emission tomography

**Fig. 6:**
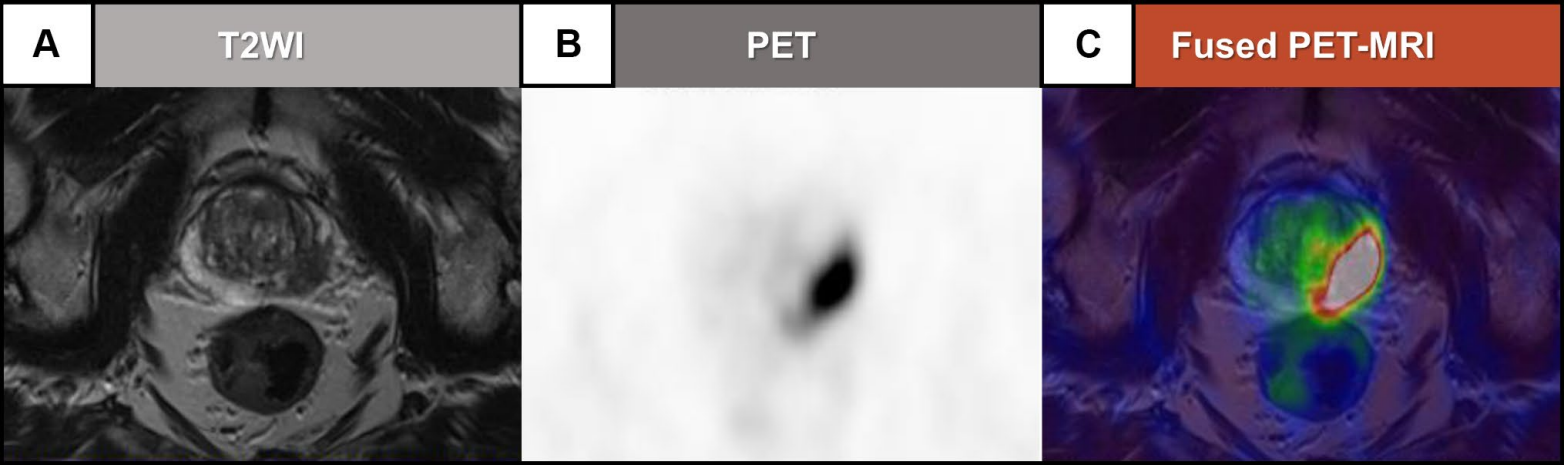
A 73-year-old man with lower urinary tract symptoms and elevated PSA levels (last one 3.7 ng/mL). Patient had a history of two previous negative transrectal biopsies, the last one performed one year before the ^68^ Ga-PSMA PET/MRI examination. The patient was submitted to a prostate ^68^ Ga-PSMA PET/MRI examination, in which T2 weighted-images (**A**) revealed a hypointense area in the left peripheral zone. PET images (**B**) demonstrated high PSMA tracer uptake in the same topography of the hypointense area on T2 weighted-images, as confirmed by fused ^68^ Ga-PSMA PET/MRI image (**C**). Image courtesy of Dr. Marcelo Livorsi da Cunha and Dr. Ronaldo Baroni from Albert Einstein Israelite Hospital, Sao Paulo, Brazil

Although the main focus of this review paper are innovations in the field of PCa MRI, it is important to cover the role of PET due to the expanding theranostic applications of PET-MRI. It is known that PSMA expression is 100–1000 times higher in malignant prostate tissue compared with benign tissue [67]. In that way, synthetic PSMA ligands have been increasingly recognized as a tool for diagnostic and therapeutic procedures in the field of PCa care. Recently, both ^68^ Ga-PSMA and ^18^F-PSMA PET/CT have been approved by the FDA for staging PCa before surgical or radiotherapy procedures, as well as to assess PCa recurrence [68]. Specifically, ^68^ Ga-PSMA PET/CT has been shown to have greater accuracy than bone and CT scanning for detecting metastatic disease, even changing cancer management in 27% of patients submitted to PSMA PET/CT that were enrolled in a prior trial [69]. In previous randomized trials, ^18^F-PSMA PET/CT was shown to have excellent specificity for detecting pelvic lymph node metastasis [70] and to have high detection rates for assessing PCa recurrence, even in patients who had low PSA levels [71]. Comparatively, although it has been shown that ^18^F-PSMA PET/CT can be superior to ^68^ Ga-PSMA PET/CT in identifying local disease recurrence and showing fewer equivocal metastatic lesions [72], data from head-to-head comparisons is still limited. Currently, both the American Urological Association and the American Society of Clinical Oncology guidelines state that ^68^ Ga-PSMA PET/CT and ^18^F-PSMA PET/CT can be indicated for patients with PCa at high risk for metastatic disease [44, 73]. Beyond PET/CT, PET/MRI has been increasingly used in recent years, offering higher soft tissue contrast and lack of ionized radiation exposure. Additionally, unlike apical PSMA expression in PCa, endothelial PSMA expression is linked with the neovascularization of benign and malignant neoplasms. In this context, MRI can be used to differentiate lesions more indicative of PCa from those of non-prostatic origin. Specifically, on a per-patient analysis, a meta-analysis that included data from 23 studies comprehending 2104 patients showed that PET/MRI had 94.9% sensitivity and 62.5% specificity to detect the primary prostatic tumor and a sensitivity of 66.7% and specificity of 93.4% to detect lymph node metastasis [74]. Figure 6 illustrates a challenging case in which ^68^ Ga-PSMA PET/MRI was useful to detect a PCa lesion. Interestingly, in this same meta-analysis, PET/MRI and PET/CT were compared in 7 of the included studies [74]. In these studies, the agreement between PET/CT and PET/MRI ranged from 71–95%. However, in 5 of 7 studies, PET/MRI was superior in detecting PCa lesions in staging and restaging, particularly for the detection of local PCa recurrences [74]. Nonetheless, a more recent systematic review and meta-analysis comprehending 8409 patients across 37 different studies showed similar detection rates of biochemically recurrent PCa for ^68^ Ga-PSMA PET/CT (70%) and ^68^ Ga-PSMA PET/MRI (71%). Therefore, large-scale randomized trials are still needed to define whether PET/MRI offers a significant benefit over PET/CT for the detection of recurrent PCa [75]. Furthermore, if proven to be of superior diagnostic value, the limited availability of PET/MRI scanners and time-consuming acquisitions, coupled with the need for technologists experienced in both nuclear medicine and MRI, as well as difficulties for accurate attenuation correction will be significant challenges for its large-scale implementation. Closer collaboration between Nuclear Medicine physicians and Radiologists during joint reading sessions will also be needed to ensure timely, comprehensive, and accurate diagnostic reports of PET/MRI examinations. The advantages and limitations of PET/CT compared with PET/MRI are summarized in Table 3.

**Table 3:** Comparison of advantages and limitations of PET/CT and PET/MRI in prostate cancer care. MRI – magnetic resonance imaging; PCa – prostate cancer

Key Factors	PET/CT	PET/MRI
Lower Acquisition times	+	−
Availability	+	−
Lower Cost	+	−
Expertise of the Operator	+	−
Lack Ionizing Radiation	−	+
Better Motion Correction	−	+
Recurrent PCa Detection Rates	−/ =	+ / =
Soft Tissue Contrast	−	+
Well defined protocols	+	−

## Notable remarks

The field of prostate MRI is constantly evolving, with the optimization of current protocols, the emergence of new sequences and devices, MRI-guided interventions, and AI algorithms. Still, the curation of current protocols adopted in clinical practice, with particular attention to prostate MRI quality should be a constant focus of diagnostic imaging practices and has been gaining significant relevance in academic debates in the last years. This is particularly demonstrated by the introduction and spread of prostate imaging quality (PI-QUAL) scoring system [54]. This tool has not only standardized an approach to objectively assess mpMRI quality, with moderate to strong interreader agreement [76–78], but also, by doing that, put the discussion of prostate MRI quality at the center of PCa research. Since its introduction in 2020, different investigations have highlighted that MRIs of lower image quality are associated with lower positive predictive value for csPCa [79], more frequent upstaging of prostate-confined PCa to locally advanced disease [80], as well as lower detection rates of PI-RADS 5 lesions and extraprostatic extension [80]. Therefore, in order to enhance image quality, practices should strive to adhere to evidence-based patient preparation protocols and align prostate mpMRI protocol according to PI-RADS recommendations. Future revisions of the PI-QUAL scoring system still need to address issues that may hinder its broader implementation, particularly in private practices. This is the case of the significant number of technical parameters listed in the PI-QUAL v1 and the lack of a scoring system to assess bi-parametric MRIs.

Another limitation of prostate MRI adhering to PI-RADS v2.1 guidelines is the agreement between different readers. Previous studies have demonstrated that interreader agreement using PI-RADS v2.1 ranges from moderate to substantial [81], with interreader agreement improving with increasing years of reader experience [82]. This creates the need for initiatives or tools that bridge this gap between less and more experienced readers. For instance, Labus et al. have shown that less experienced readers when assisted by a deep learning algorithm had a significant increase in the area under the cover to detect csPCa, from 0.68 to 0.80. In this same study, the authors demonstrated that the area under the curve of less experienced readers assisted by the deep learning algorithm was similar to that of experienced readers not using the deep learning algorithm (0.80 and 0.81, respectively) [83]. This exemplifies how innovations in prostate MRI, such as AI algorithms, may contribute to enhancing the accuracy of prostate MRI reports and the agreement between readers, particularly for those with less experience.

Another important point to consider for invasive innovations is that, as for any procedure, MRI-guided focal therapy and radiotherapy interventions in prostate cancer treatment are subject to a learning curve [84, 85]. Blazevski et al.’s study exemplifies this, showing that after irreversible electroporation, 78% of men were free of clinically significant prostate cancer (csPCa) at 12 months. However, excluding the initial 32 patients undergoing irreversible electroporation increased this number to 85% free of csPCa [86]. Therefore, it is crucial to consider the learning curve associated with interventional, as initial cases may show lower effectiveness than expected. This should also be taken into consideration in clinical practice, emphasizing the need for ongoing training and expertise of physicians using these novel techniques.

## Conclusion

Several innovations in the field of PCa care, spanning from refinements in current MRI modalities to novel diagnostic and MRI-guided procedure applications as well as AI algorithms, hold promise in improving patient outcomes and reducing the need for invasive diagnostic and therapeutic procedures. Nevertheless, robust evidence from large-scale multicenter prospective studies will define which innovations are ready for prime time and the ones that are no more than a passing fad.

## Supplementary Information

Below is the link to the electronic supplementary material.

Supplementary file1 (MP4 9491 KB)
